# Promoting health education and public awareness about dengue and its mosquito vector in Saudi Arabia

**DOI:** 10.1186/s13071-014-0487-5

**Published:** 2014-11-18

**Authors:** Al Thabiani Aziz, Salman A Al-Shami, Jazem A Mahyoub, Mesed Hatabbi, Abu Hassan Ahmad, Che Salmah Md Rawi

**Affiliations:** Department of Biology, Faculty of Science, University of Tabuk, Tabuk, 71491 Saudi Arabia; Department of Biological Sciences, Faculty of Science, King Abdulaziz University, P. O. Box, 80203, Jeddah, 21589 Saudi Arabia; Ministry of Health, Jeddah, Kingdom of Saudi Arabia; School of Biological Sciences, Universiti Sains Malaysia, Penang, 11800 Malaysia

**Keywords:** Dengue, Health education, Public awareness, *Aedes aegypti*, KSA

## Abstract

Currently, dengue fever is considered as the main health problem in several parts (Mekkah, Jeddah, Jazan and Najran) of Kingdom of Saudi Arabia (KSA) with dramatically increase in the number of cases reported every year. This is associated with obvious ineffectiveness in the recent control and management programs for the mosquito vector (*Aedes aegypti*). Here, we suggested promoting the health education and public awareness among Saudi people to improve the control of dengue mosquito vector. Several suggestions and recommendations were highlighted here to ensure effectiveness in the future control and management programs of dengue mosquito vector in KSA.

## To the Editor

It was very interesting to read the recent letter of Arya and Agarwal [1] as an apropos to the earlier paper published by Aziz et al. [2]. In this regard, we would like to highlight the importance of health education about dengue and its mosquito vector in Saudi Arabia.

It would be important to emphasize here that health education and personal sanitation are necessary initial steps in modern control of mosquito borne as it involves removal of possible breeding sites of larvae. Unfortunately, such strategies are rarely experienced in the Kingdom of Saudi Arabia (KSA). Thus, awareness of dengue vector life cycle and its preferable domestic and peri-domestic habitats is almost absent. In the same context, developing the public awareness and promoting the public health should be conducted along with larval and adult control strategies.

For dengue control, public awareness and health education regarding the habitat and life cycle of the mosquito vector, as well as its physical and cultural control, are important in population management. It is acceptable fact that there is severe lacking in the awareness as well as health education about dengue and its mosquito vector among the residents in KSA. Despite that, there are very few studies that have highlighted the importance of health education for reducing the dengue cases in KSA [3].

Health education is proven to be an essential step in any vector control program which implies sustaining efficient information and scientific knowledge to the society on transmitted diseases and their vectors. The knowledge on the vector life cycle and its ecology and biology should be delivered to help the people to live in a healthy conditions and destruction of vectors breeding sites.

Therefore, the following recommendation are suggested in order to improve the public awareness and health education on dengue and its mosquito vector in KSA:

- Public awareness should be promoted for prevention and control the dengue fever. This should involve cooperation between private and public sectors.
- Health education strategies should educate the people to break the mosquito life cycle by destroying the possible mosquito breeding sites such as concrete pools, water tanks, aquaria, irrigation ditches and drainages as well as air-conditioners and disposable tires.
- The immediate-action principle should be activated associated with effective communication and cooperation between different government sectors. Positive dengue cases should be followed by intensive management of the vector in the possible breeding sites where the case came from.
- Enhancing self-awareness among the people through health education programs. Different activities should be organized including practicing self-protection and regular workshops on the larvae and adults control strategies. It is important to educate people about the adverse effects of the arbitrary application of insecticides without prior knowledge on dose, resistance and side effects of these chemicals.
- It is suggested that Saudi Ministry of Health as well as Ministry of Municipals should launch different workshops to increase the knowledge about dengue and biology and ecology of the mosquito vector in the society. Regular education programs should be conducted out at different levels to highlight the importance of personal sanitation in preventing dengue.
- The knowledge, attitude and practice (KAP) on dengue and its mosquito vector should be assessed and improved among Saudi people.
- The residents should be aware not merely to control activities carried out by the control personnel but also interacting with health education organized by the respective authorities and government bodies. The experience in application of health education programs in other dengue endemic countries such as India, Malaysia and Singapore should be adopted in KSA.
- The Saudi Ministry of Culture and Information should establish intensive health education programs through producing the announcements and advertisements in radio, press and television to increase the public awareness about the dengue and its vector control as well as how to maintain hygiene conditions inside the houses.
- The Destruction of Disease-Bearing Act (DDBA) should be enforced in KSA to control the population of dengue vectors.
